# Association between cesarean section and labor companion in pregnant women giving birth: A prospective cohort study at Hung Vuong hospital, Vietnam

**DOI:** 10.18332/ejm/219763

**Published:** 2026-04-21

**Authors:** Tuan Minh Vo, Thien Tran Minh Ngo, Nam Hoai Nguyen, Du Van Tran, Dat Quoc Nguyen, Hung Minh Tran, Tuyet Thi Anh Hoang

**Affiliations:** 1 Faculty of Medicine, University of Medicine and Pharmacy at Ho Chi Minh City, Ho Chi Minh City, Vietnam; 2 Hung Vuong Hospital, Ho Chi Minh City, Vietnam

**Keywords:** active labor phase, cesarean section rate, counseling, labor companion, pregnancy outcome

## Abstract

**INTRODUCTION:**

The American College of Obstetricians and Gynecologists (ACOG) suggests certain measures to decrease the cesarean section rate, including the use of a labor companion model for pregnant women. The objective of this study is to evaluate and compare cesarean section rates among women with labor companions versus those without, in one of the biggest hospitals in Vietnam.

**METHODS:**

A prospective cohort study was conducted on 394 pregnant women at Hung Vuong Hospital, Vietnam, between December 2023 and January 2024. The study participants were recruited into two groups: 197 women who had labor companions and 197 women who did not. Multiple regression analysis, with a 95% confidence level, was applied to investigate the association between labor companionship and cesarean section rates.

**RESULTS:**

The rate of cesarean sections was significantly lower in women who had a labor companion, 29.4% compared to 41.6% in those without a companion. The presence of a labor companion was associated with a lower risk of cesarean sections (adjusted risk ratio, ARR=0.65; 95% CI: 0.46–0.91), and it also led to an increased use of obstetric anesthesia (p<0.05).

**CONCLUSIONS:**

The findings suggest that the presence of a labor companion is associated with a lower cesarean section, and healthcare providers may consider this during counseling sessions with patients.

## INTRODUCTION

Pregnant women often receive care and support during labor and delivery from trusted individuals who are familiar with their needs. These caregivers, who may be family members or community birth attendants, provide emotional encouragement and support during the labor process, a concept known as ‘continued support’^1^. However, these caregivers typically lack medical knowledge about reproduction, drawing from their own experiences instead. To ensure the safety of both mother and newborn, many pregnant women now prefer to have their labor managed in healthcare facilities rather than at home, despite the benefits of ‘continued support’ being disrupted^2^. This has sparked a push to integrate the medical expertise of healthcare systems with the emotional support provided by companions. In line with this goal, guidelines and recommendations from the World Health Organization (WHO) emphasize the importance of having companions present during labor. These companions can be chosen by the pregnant woman and may include family members, close friends, colleagues, and medical staff^1,2^. The World Health Organization (WHO) suggests that the cesarean section rate should not exceed 10–15% of live births. However, this rate is currently increasing globally^3^. According to global data from 2018, the cesarean section rate stood at 21.1%, with a significantly higher rate of 44.9% recorded in East Asia^4^. In Vietnam, the cesarean section rate has reached 49.6%, with private hospitals reporting a rate of 57.8% and public hospitals at 49.1%^5,6^. Reducing the cesarean section rate is a pressing issue for obstetrics hospitals. WHO has released new guidelines emphasizing the importance of a companion during labor, which can help reduce cesarean section rates, shorten labor duration, and improve newborn outcomes^7^. A labor companion can be a family member, such as a husband, birth mother, or in-laws, or a trained assistant. Despite the benefits of labor companions, the WHO’s recommendation has not been widely adopted in Vietnam due to various challenges, including infrastructure limitations, medical staffing shortages, and patient-related issues^8^.

Hung Vuong Hospital is a tertiary-level obstetrics and gynecology facility that manages over 40000 live births annually, with the spontaneous birth-rate fluctuating between 50–54% on average^9,10^. The hospital’s birth delivery department has two main service areas: one for standard services and another for those who request additional support. Due to limited infrastructure, labor companions for expecting mothers are currently only available in the requested service area and may not be present throughout the entire labor process. Within this setting, a chosen family member would be advised by administrative nurses before entering the delivery room on what to do and not to do: 1) Assist in wearing protective clothing including gowns, hats and medical masks correctly; 2) Things to do for pregnant women, sit next to them and hold their hands, ask about their well-being and offer encouragement, help them change positions or give them massages, give them water as needed, help mediate positively in conveying information between pregnant women and medical staff, and make decisions with the consent of family members in a timely manner; and 3) do not touch or operate medical equipment or medications, do not perform or interfere with medical instructions, and do not leave the area without permission.

Research suggests that having a family member present can provide emotional support to an expecting woman, helping alleviate feelings of loneliness, anxiety, and fear during labor^7,8^. However, technical and infection control requirements are significant barriers to expanding this model to more expectant mothers. Furthermore, a meta-analysis published in 2024^11^ indicated that there is insufficient evidence to define the ‘best labor companion’ owing to the heterogeneity of labor companions and outcome assessment across different studies. The meta-analysis concluded that well-designed further research is warranted to fill this gap.

This study aims to evaluate the feasibility of scaling up the labor companion model at Hung Vuong Hospital by examining its impact on cesarean section rates.

## METHODS

### Study design and sample size

A prospective cohort study was carried out at Hung Vuong Hospital during the period December 2023 – January 2024. Eligible pregnant women for the study included those with a gestational age of 37 weeks or more and in the active labor stage with a cervical opening of 4 cm or more. However, exclusion criteria included women under 18 years old, those with mental illnesses or unable to communicate in Vietnamese, stillbirth cases, and those without a first-trimester ultrasound to determine gestational age or pre-indicated for cesarean section. Participants who met the criteria and agreed to participate were assigned to either a group with a labor companion or one without a companion.

The sample size was calculated with the formula comparing two proportions. As per the Dubey et al.^12^ study, cesarean section rates in the two groups were: p1=10.7% and p2=21.3%, respectively. Therefore, the minimum sample size per group should be n1= n2=187. Loss to follow-up was expected for 5%, and hence, the estimated sample size per group was 197 pregnant women, and the total sample size was 394.

### Data collection tools

The researchers employed a pre-formatted interview questionnaire containing 23 questions to gather data on epidemiology, medical history, and pregnancy outcomes prior to conducting laboratory tests.

### Primary variables

The presence of a labor companion, often a family member, is to provide emotional and physical support to a pregnant woman during labor. This support includes offering reassurance and encouragement to calm her, as well as physical assistance such as massage, respiratory guidance, and help with changing positions. Labor companions also serve as a link between the pregnant woman and medical staff, enabling them to communicate effectively and alert medical personnel to any issues that may arise during labor. A labor companion’s involvement was determined by the question: ‘Do you have a companion during the labor process?’. The answer was recorded in the interview questionnaire. The delivery method was also noted in the patient’s medical records.

### Study process

*Sampling*

Not all pregnant women have family members with them during labor, as this is limited only to facilities that charge extra fees. Even in premium facilities, the presence of a family member is at the pregnant woman’s request, as she can choose which family member joins her during active labor when she moves to the delivery room. Traditionally, she can change her companion in the delivery room, but our study found no instances of such changes.

After meeting the inclusion criteria, participants were informed about the study’s goals and signed a consent form in the waiting room. Those who consented were advised about having a companion during active labor. Their information, including name, ID, and companion status, was recorded. A midwife provided education to the companion, typically a family member or partner, in the waiting area. Furthermore, the counseling team distributed leaflets to companions to remind them of what to do and what not to do when with the pregnant woman during labor. Three pregnant women were selected, by convenience sampling, from daily admission records for each study group.

*Pilot study*

A mock interview was conducted with 5 pregnant women in December 2023 to assess the suitability of the interview questionnaire and labor recorder. Participants consisted of 3 women who gave birth with a companion and 2 who did so without. All 5 pregnant women found the questionnaire clear and preferred the study. The information collected during the trial was easily understood and did not disrupt the daily activities of medical staff at the delivery department. The 5 pregnant women who participated in the trial were not included in the final study sample for the purpose of maintaining objectivity.

*Data collection*

After childbirth, pregnant women were transferred to other departments for ongoing care until discharge. Following post-delivery guidelines at Hung Vuong Hospital, women who gave birth vaginally were monitored for 3 days before being discharged. Women who underwent cesarean sections received care for 5 days before discharge. The researcher matched study participants with patient transfers from delivery or resuscitation departments to identify their destinations for aftercare. Interviews were conducted with participants the day after delivery, typically at 9:00 am, after they had undergone a medical check. If a participant was absent or unavailable, the researcher returned in the afternoon or the following day, usually at 3:00 pm.

If a participant wished to withdraw or was unavailable for a face-to-face interview due to her post-delivery status, she was removed from the study sample. A substitute pregnant woman was then screened for eligibility to participate in the study. Following hospital discharge or transfer, the researcher retrieved information on labor progress and newborn outcomes from medical records.

### Ethical considerations

This study was conducted in accordance with the Helsinki Declaration of 1975, as revised in 2024. Ethics approval for the study was obtained from the Institutional Review Board of the University of Medicine and Pharmacy at Ho Chi Minh City (No: 6653/HDDD-BVHV, 11 November 2023). Informed consent was obtained from all patients. Data were kept anonymous and confidential during all stages of the study.

The reporting of this study conforms to the STROBE guidelines^13^.

### Statistical analysis

Data analysis was performed utilizing the Stata 17.0 software (Stata Corp LLC, Lakeway Drive, College Station, April 2021, TX, USA). Continuous variables were summarized with mean values and standard deviations, whereas categorical variables were presented as proportions and percentages. Poisson regression models, both univariate and multivariate, were employed to investigate the relationship between the cesarean section rate and the independent variables. The independent variables involved in the final multivariable regression included exposure variables and potential covariates. A potential covariate was included in the final multivariable logistic regression model if its association with the outcome in bivariate analysis had a p≤0.2. Adjusted risk ratios (ARRs) and their 95% confidence intervals are reported.

## RESULTS

**Figure 1:**
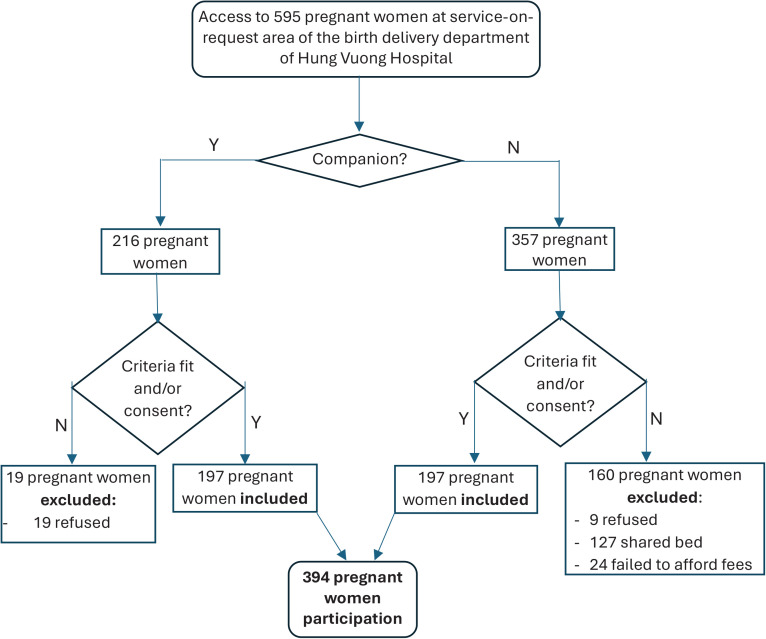
Sampling process flowchart

Within the designated service area, researchers approached 237 pregnant women accompanied by someone and 358 women without companions. After providing information on the benefits and rights of participating in the study, a total of 394 pregnant women and their relatives voluntarily agreed to participate. The study involved two equal groups, each consisting of 197 pregnant women – those with companions and those without (Figure 1). The participants were registered on a study list, and the data collection process was initiated.

The study’s participants’ social characteristics are outlined in Table 1. The average age of participants was 28.7 years with a standard deviation of 4.5 years and an age range of 18 to 42 years. Most participants engaged in housework, accounting for 48.5% of the total. Senior high school and higher education levels were prevalent among participants, comprising 84% of the group. Approximately two-fifths of participants resided in urban areas. No significant differences were observed in demographics (age, occupation, education level, residence, economic status) (all p>0.05).

**Table 1:** Demographic characteristics, by labor companion status, of pregnant women giving birth at Hung Vuong Hospital, Vietnam, December 2023 – January 2024 (N=394)

*Characteristics*	*Total (N=394)* *n (%)*	*Companion*		*p*
		*No* *(N=197)* *n (%)*	*Yes* *(N=197)* *n (%)*	
**Maternal age** (years), mean ± SD (range)	28.7 ± 4.5 (18–42)	28.3 ± 4.3 (19–2)	29.0 ± 4.3 (18–39)	0.32^a^
<20	9 (2.3)	2 (1.0)	7 (3.6)	0.11^b^
20 to <35	345 (87.6)	171 (86.8)	174 (88.3)	
≥35	40 (10.1)	24 (12.2)	16 (8.1)	
**Occupation**				
Housework	191 (48.5)	91 (46.2)	100 (50.8)	0.87^b^
Manual work	85 (21.6)	44 (22.3)	41 (20.8)	
Intellectual work	118 (29.9)	62 (31.5)	56 (28.4)	
**Education level**				
Junior high and lower	67 (17)	41 (20.8)	26 (13.2)	0.09^c^
Senior high	93 (23.6)	41 (20.8)	52 (26.4)	
Higher education	234 (59.4)	115 (58.4)	119 (60.4)	
**Residence**				
Urban	172 (43.7)	87 (44.2)	85 (43.2)	0.70^c^
Suburban	63 (16.0)	34 (17.3)	29 (14.7)	
Other	159 (40.3)	76 (38.6)	83 (42.1)	
**Income**				
Poor	28 (7.1)	14 (7.1)	14 (7.1)	0.40^c^
Middle	193 (49.0)	103 (52.3)	90 (45.7)	
High	173 (43.9)	80 (40.6)	93 (47.2)	

a Chi-squared test.

b Fisher’s test.

c t-test.

There were no notable differences in demographics (age, occupation, education level, residence, economic status), as all p-values were above 0.05.

The obstetric history of the participants is depicted in Table 2.

**Table 2:** Obstetric history of pregnant women, by labor companion status, giving birth at Hung Vuong Hospital, Vietnam, December 2023 – January 2024 (N=394)

	*Total* *(N=394)* *n (%)*	*Companion*		*p*
		*No* *(N=197)* *n (%)*	*Yes* *(N=197)* *n (%)*	
**Parity**				
Primiparous	278 (70.6)	131 (66.5)	147 (74.6)	0.08^a^
Multiparous	116 (29.4)	66 (33.5)	50 (25.4)	
**Full-term birth**				
No	282 (71.6)	133 (67.5)	149 (75.6)	0.07^a^
Yes	112 (28.4)	64 (32.5)	48 (24.4)	
**Premature birth**				
No	388 (98.4)	194 (98.5)	194 (98.5)	1.00^b^
Yes	6 (1.6)	3 (1.5)	3 (1.5)	
**Previous cesarean section**				
No	389 (98.7)	193 (98.0)	196 (99.5)	0.37^b^
Yes	5 (1.3)	4 (2.0)	1 (0.5)	
**Miscarriage/abortion/ectopic/molar pregnancy**				
None	312 (79.2)	153 (77.7)	159 (80.7)	0.80^a^
1	67 (17.0)	36 (18.3)	31 (15.7)	
≥2	15 (3.8)	8 (4.0)	7 (3.6)	
**Prenatal BMI**				
Underweight	64 (16.3)	27 (13.7)	37 (18.8)	0.14^a^
Normal	261 (66.2)	129 (65.5)	132 (67.0)	
Overweight	63 (16.0)	36 (18.3)	27 (13.7)	
Obese	6 (1.6)	5 (2.5)	1 (0.5)	
**Prenatal weight** (kg), mean ± SD (range)	65.5 ± 8.3 (43–95)	65.9 ± 8.9 (47–95)	65.1 ± 7.7 (43–87)	0.34^c^
**Number of antenatal visits**, mean ± SD (range)	13.3 ± 2.9 (4–25)	13.2 ± 2.7 (5–20)	13.3 ± 3.0 (4–25)	0.73^c^
**Antenatal care at a private clinic**				
No	125 (31.7)	65 (33.0)	60 (30.5)	0.59^a^
Yes	269 (68.3)	132 (67.0)	137 (69.5)	
**Antenatal care at the hospital**				
No	171 (43.4)	83 (42.1)	88 (44.7)	0.61^a^
Yes	223 (56.6)	114 (57.9)	109 (55.3)	

a Chi-squared test.

b Fisher’s test.

c t-test.

Table 3 presents the univariate analysis of labor companion factors and cesarean section rates. In univariate analyses, the labor companion group had a reduced risk of cesarean section, 0.71 (95% CI: 0.50–0.99), compared with the group without a companion (p<0.05).

**Table 3:** Relationship between companion and cesarean section, by labor companion status, of pregnant women giving birth at Hung Vuong Hospital, Vietnam, December 2023 – January 2024 (N=394)

*Description*	*Birth method*		*RR*	*95% CI*	*p*
	*Cesarean section* *(N=140)* *n (%)*	*Vaginal delivery* *(N=254)* *n (%)*			
**Companion**					
Yes	58 (29.4)	139 (70.6)	0.71	0.50–0.99	**0.04**
No (ref.)	82 (41.6)	115 (58.4)			

RR: risk ratio.

Seventeen independent variables were used for the univariate analysis. The dependent variable was cesarean section; independent variables included: having a companion, pain relief during labor, and the 15 other variables listed in Tables 1 and 2. To identify factors associated with cesarean section, univariate regression analyses were conducted first. Associations between cesarean section and 17 independent variables were examined. Five variables were significantly associated with cesarean section (p<0.05). These five independent variables were included in a multivariate analysis model aiming to control for covariates and obtain a better adjusted association between companionship and cesarean section rate (Table 4). Within the adjusted multivariate regression analysis, the group with labor companions was noted to have a reduced risk of having performed a cesarean section (ARR=0.65; 95% CI: 0.46–0.91; p<0.05) compared with the no-companion group.

**Table 4:** Relationship between companion and cesarean section, by labor companion status, of pregnant women giving birth at Hung Vuong Hospital, Vietnam, December 2023 – January 2024 (N=394)

*Variables*	*Birth delivery method*		*RR*	*ARR*	*95% CI*	p*
	*Cesarean section* *(N=140)* *n (%)*	*Vaginal delivery* *(N=254)* *n (%)*				
**Companion**						
Yes	58 (29.4)	139 (70.6)	0.71	0.65	0.46–0.91	0.01
No (ref.)	82 (41.6)	115 (58.4)				
**Occupation**						
Manual work	26 (30.6)	59 (69.4)	1.02	1.09	0.68–1.73	0.73
Intellectual work	57 (48.3)	61 (51.7)	1.62	1.54	1.07–2.23	0.02
Housework (ref.)	57 (29.8)	134 (70.2)				
**Parity**						
Multiparous	17 (14.7)	99 (85.3)	0.33	0.71	0.1–5.15	0.74
Primiparous (ref.)	123 (44.2)	155 (55.8)				
**Full-term birth**						
Yes	16 (14.3)	96 (85.7)	0.32	0.53	0.07–3.98	0.53
No (ref.)	124 (44.0)	158 (56.0)				
**Obstetric anesthesia**						
Yes	125 (39.7)	190 (60.3)	2.09	1.78	1.03–3.08	0.04
No (ref.)	15 (19.0)	64 (81.0)				

RR: risk ratio by univariate regression. ARR: adjusted risk ratio by multivariate regression.

* By multivariate regression.

## DISCUSSION

Within our study, all labor companions received the same counseling and guidance; the study did not assess how the impact of accompanying people varied during labor, but focused simply on whether the presence or absence of a companion during labor affected the cesarean section rate.

An adjusted RR of 0.65 corresponds to a 35% lower relative risk. The risk of cesarean section was reduced by 35% in the group with a labor companion compared to those without a companion. This finding is similar to a 2017 meta-analysis conducted by Bohren et al.^14^, which analyzed data from over 15000 pregnant women across 17 countries. In that analysis, Bohren et al.^14^ focused-on companions who provided emotional support, such as reassurance, encouragement, and updates on labor progress, during the labor process. The results found that pregnant women with a labor companion were less likely to have a cesarean section rate (0.75; 95% CI: 0.64–0.88) compared to the no-labor-companion group. A study conducted in New Delhi^12^, India, from 2019 to 2021 analyzed the impact of labor companions on pregnant women. Researchers chose 150 pregnant women and randomly divided them into two groups: one with labor companions and the other without. The study found a cesarean section rate of 21.3% in the group without labor companions and 10.7% in the group with labor companions. The presence of a labor companion resulted in a significant reduction in the likelihood of a cesarean section (p<0.05).

Contrary to previous findings, Hodnett et al.^15^ discovered that cesarean section rates were 12.5% in the companion group and 12.6% in the no-companion group, with no significant difference between the two. However, the results of our study may differ due to the types of companions involved – specially trained midwives in their study versus close family members in ours. The concept of a labor companion appears to be more commonly applied to pregnant women’s close relatives, such as husbands or family members, rather than medical staff.

Another aspect to consider is obstetric anesthesia, with only 80% of participants requesting it for labor. This significant demand can be attributed to the preference of expectant mothers who chose specific service areas for childbirth, regardless of whether they had a support person present. At Hung Vuong Hospital, obstetric anesthesia typically involves epidural techniques using anesthetics like lidocaine. This method serves as both an anesthetic option for cesarean deliveries and a primary means of pain relief for vaginal births. The presence of a labor companion has been shown to enhance the utilization of obstetric anesthesia, as this close relative often empathizes with the woman’s pain and encourages her to consent to the anesthesia. Consequently, in our study, the involvement of a labor companion was associated with an increase in the demand for obstetric anesthesia compared to those without a companion. Our study findings indicated that the presence of a companion during labor can be advantageous, leading to a 35% decrease in the likelihood of cesarean deliveries. These results hold important implications for how labor and support care are approached, especially during the crucial active labor stage. When a close family member is available during labor, healthcare professionals might be able to reduce cesarean section rates and enhance overall pregnancy outcomes. In addition, the evidence gained from this research could influence how medical personnel view and engage with the concept of companionship during labor in Vietnam.

### Limitations

This research has certain limitations that could influence its effectiveness. First, the use of convenience sampling and a non-random allocation of exposure may lead to selection bias, especially when assessing two different groups. Although potential confounding variables were examined, no significant relationships were found. Second, this research is solely observational, and the duration of companion attendance varied among participants without any form of regulation. Third, while the presence of a companion during labor was noted, there was no specific evaluation of their influence or interaction with each pregnant woman at various levels. This assessment is essential for improving companion counseling.

## CONCLUSIONS

The research indicates that the presence of a companion during labor significantly decreased the likelihood of a cesarean section in our setting. To further validate these findings, additional studies should be conducted to examine the effectiveness and support of companion presence during the entire labor process, including the latent phase. Our results imply that it is important to educate pregnant women about the benefits and impacts of having a labor companion within our setting. However, we note that labor companions need to be provided with specific guidance on their role, responsibilities, and the support they can offer to the pregnant woman during the labor process.

## Data Availability

The data supporting this research are available from the authors on reasonable request.
